# How Do Children of Parents With Mental Illness Experience Stigma? A Systematic Mixed Studies Review

**DOI:** 10.3389/fpsyt.2022.813519

**Published:** 2022-02-18

**Authors:** Lisa-Marie Dobener, Julia Fahrer, Daniel Purtscheller, Annette Bauer, Jean Lillian Paul, Hanna Christiansen

**Affiliations:** ^1^Department of Clinical Child and Adolescent Psychology, Philipps University Marburg, Marburg, Germany; ^2^Mental Health Research Program, The Village, Ludwig Boltzmann Gesellschaft, Innsbruck, Austria; ^3^Department of Psychiatry, Psychotherapy and Psychosomatics, Division of Psychiatry I, Medical University Innsbruck, Innsbruck, Austria; ^4^Care Policy and Evaluation Centre (CPEC), London School of Economics and Political Science, London, United Kingdom

**Keywords:** children of parents with mental illness, stigma, child mental health, systematic review, qualitative content analysis

## Abstract

Stigma can have devastating health and wellbeing impacts, not just on people with mental health problems, but on people associated with the stigmatized person. This is called stigma-by-association. Children whose parents have mental health problems are a particularly vulnerable group, and stigma acts as a mechanism, contributing to the transgenerational transmission of mental disorders. The current study is a systematic mixed studies review, synthesizing knowledge about how this group of children experience stigma-by-association. Overall, 32 studies were included, after a systematic search including quantitative, qualitatative, and mixed methods studies. The methodological quality was assessed and qualitative content analysis undertaken. We grouped children's stigma experiences into four dimensions, i.e., experienced stigma, anticipated stigma, internalized stigma, and structural discrimination. Results show that stigma is an important factor in those children's lives, and needs further investigation in qualitative and quantitative research. The current study emphasizes the importance of anti-stigma interventions and campaigns.

## Introduction

Children of parents with a mental illness^1^ have received increasing attention over recent years, especially as it is estimated that one in five children world-wide has a parent with a mental illness (1–3). Those children often face specific challenges that are associated with reduced mental health, poorer academic achievement, and impaired social well-being and quality of life (1, 4, 5). Social adversities associated with mental illness, including poverty, as well as genetic predisposition and family dysfunction, act as mechanisms contributing to these risks (6). Those mechanisms have been integrated into a comprehensive model: the transgenerational transmission of mental disorders (TTMD) model identifies four major domains (1. parent, 2. family, 3. child, 4. social environment) that interact with their respective systems, and are influenced by five transmission mechanisms^1^ (1. genetics, 2. prenatal factors, 3. parent-child-interaction, 4. family, and 5. social factors) (1, 7). According to this model, stigma can be seen as a component of social factors, acting as a mechanism for the transmission of mental disorders that leads to multiple challenges and negative outcomes.

As captured in the concepts of stigma-by-association (SBA) (8), family stigma [e.g., (9)], or courtesy stigma (10, 11), stigma can affect not only the person with a mental health problem, but also people connected to the person, such as family or friends (12). The conceptual thinking of SBA by Pryor et al. (8) focuses on the general public and potential mechanisms that might contribute to the emergence of SBA, but lacks the perspective and experiences of those affected. Current research mostly describes SBA as guilt, blame, and contamination, ascribed to and experienced by family members (13, 14). Children are most frequently described as experiencing such “contamination” stigma, i.e., the general public tends to see them as being contaminated by the parental mental illness [e.g., (13, 14)]. The theoretical model of SBA by Philipps and Gates (15) focuses on various stigma dimensions and facets that could affect children, but for the very special group with incarcerated parents. On an experiential level, perceived stigma, internalized stigma, discrimination, and differences in social, cultural, economic, and political power are identified for this group. Another qualitative study classified stigma dimensions described by family members of people with schiziophrenia, finding structural elements of discrimination, and interpersonal interaction elements, such as social exclusion (16, 17).

Goffman has defined experiencing “courtesy stigma” as ‘an individual who is related through the social structure to the stigmatized (…) leads the wider society to treat both individuals in some respect as one' (11), and research so far has not conceived of the stigma dimensions for relatives, but rather solely named the stigma facets to be important for relatives of people with a stigmatized condition. Given this, we assume that SBA for children can potentially contain all of the stigma facets described for the primary recipients: *Experienced SBA* describes personally experienced prejudice and discrimination (18, 19); *perceived SBA* explains the “belief that most people will devalue, discriminate the stigmatized” (19); *anticipated SBA* includes expectations that others will devalue and discriminate against them (18); *affiliate stigma* describes the self-stigma of associates of people with a mental illness, i.e., the “internalization of stigma among associates of targeted individuals” on the affective, cognitive and behavioral level (20); and *structural discrimination* targets societal and policy structures that reproduce existing social inequalities (21).

The experience of SBA and discrimination, the anticipation of what others may think about them, and accommpaning self-stigmatization, can have a deep impact on individuals. There is evidence that 8–22 % of family members of people with a mental illness experienced stigma's negative impacts on themselves, e.g., ruined self-esteem (21%), disrupted family relationships with other family members (22%), and with their ill family members (20%) (16). It has also been reported that family members of someone with a mental illness may avoid social situations and events, reduce or break contact with family and friends, spend energy on hiding the secret, and experience discrimination within employment and/or housing situations (14, 22). Although a growing body of research focuses on SBA, very few studies investigated how these stigma experiences affect individual family members (23); systematic studies, especially those targeting children, are missing that would shed light on their SBA experiences while classifying their experiences into the various stigma dimensions. The same is true for the concept of affiliate stigma (20): while children of parents with a mental illness often do care for their parents [parentification, e.g., (24)], they have not been the focus in studies on affiliate stigma so far.

A recent systematic review on the evidence of stigma concepts for children of parents with a mental illness aimed to identify stigma-related experiences and outcomes as reported by parents and children (25). Their findings summarize stigma concepts from the primary literature, all of which describe different individual facets of children's and their parents' experiences of stigma. This review highlights the lack of uniform definitions for such stigma experiences, as well as the lack of an all-encompassing concept that includes the various dimensions of stigma experiences of children whose parents have a mental illness. The main finding of this review is that affected children report feelings of embarrassment, shame, and the need to hide their parental mental illness, though those findings are not integrated into an overall framework of different stigma dimensions.

The aim of the present study is therefore (1) to gain knowledge about how the children of parents with a mental illness experience stigma, (2) to synthesize this knowledge into a primary model of SBA on an experienced level for this population. In so doing, we (3) hope to contribute to SBA's theoretical model, that is currently insubstantial and not focused on children.

To achieve this, evidence from qualitative, quantitative, and mixed-method studies regarding children's stigma experiences related to their parents' mental illness is synthesized and collated.

## Methods

### Protocol and Registration

The review is registered and approved by PROSPERO, registration number CRD42019112838 (www.crd.york.ac.uk/PROSPERO).

### Design

This review follows the PRISMA statement for conducting systematic reviews (26). Considering stigmatization of mental illness and its impact upon children is a complex and multifaceted problem. We thus included qualitative, quantitative, and mixed-methods studies to synthesize knowledge from different methodological perspectives, and diverse evidence.

### Search Strategy

**Figure 1:**
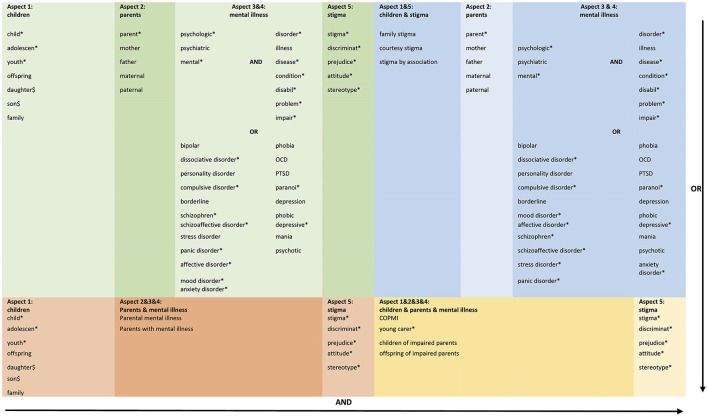
Search strategy.

Five electronic databases (PubMed, Cochrane Library, PsycINFO, PSYNDEX, Web of Sciences) were searched (updated April 2020), using a detailed search strategy developed for PubMed and adapted for other databases, see Figure 1. The four different search term combinations (illustrated in the figure by the colors blue, green, orange and yellow) were combined with the OR function. Additional references from current reviews and theoretical articles were also reviewed to identify additional citations. Our search strategy consisted of five aspects and was developed with the Village research team (https://village.lbg.ac.at/about), an expert librarian, and an expert in stigma theories.

### Eligibility Criteria

Studies were eligible for inclusion if they met the following criteria: (a) original primary peer-reviewed research; (b) published in English or German, (c) investigation of stigma experiences, or stigma as a relevant issue for children, and (d) children of parents with a mental illness were a (sub-)sample in the study. We did not use a filter for publication dates.

### Exclusion Criteria

Studies were excluded if they: (a) focused on the general public as study population, i.e., we were not interested in the stigma that public stigma carries against children, but on the experience-level of the affected individuals, or (b) their stigma measures did not allow for a clear distinction between the different stigma dimensions, or if their only informative value for children was whether they experienced more or less stigma than other family members, or (c) did not differentiate enough between children and other family members regarding their stigma experiences, i.e., if the original paper did not state clearly if their results reflect the experiences of all family members, including children, we excluded them as we could not ensure whether those findings applied to this population. In qualitative studies with different subsamples, we used only those statements made by the children. In quantitative studies, we only used the analysis results of the subgroup of children.

### Study Selection

After conducting the standard search as outlined above, all titles and abstracts were screened by independent reviewers (L-MD, FS). They were eligible for full text screening if children of parents with mental illness were participants in the study, and if the abstract either directly highlighted stigma or stigma-related concepts (see search strategy, aspect 5) or included children's specific experiences that may include stigma experiences. Full texts of potentially eligible studies were retrieved and independently assessed for eligibility by two review authors (L-MD, JF). Any disagreement was resolved through discussion with a third review author (HC).

### Quality Appraisal

Three review authors (L-MD, AB, JLP) independently assessed the risk of bias in the included studies. Two authors independently appraised the quality and looked for convergence. Due to heterogeneity in methodological approaches in the included studies, i.e., qualitative, quantitative as well as mixed methods methodology, the “Mixed Methods Appraisal Tool” [MMAT; (27)] was used. The quality of each study was assessed applying two general criteria for all the studies, and five criteria adapted to each specific methodology requirement. As recommended by the authors of the MMAT tool, we did not exclude any studies due to low methodological quality, but used their information to estimate potential biases. Nevertheless, to provide an overview of study quality, we calculated an overall score for each study in terms of study quality, ranging from 0 to 100%, in which we allocated one star for each 20% of the criteria met.

### Data Extraction

Various information was extracted from the original studies addressing our research question. In addition to general information on data collection, analysis methods, and study population, all text passages related to stigma experiences were extracted. This means that the results and discussion sections of the included studies were systematically searched for stigma-related terms and content. Once the data analysis was completed, we determined which stigma dimensions could be allocated to which studies. All the extracted data are summarized in Supplementary Table S1. Two authors (L-MD, DP) extracted data independently, discrepancies were identified and resolved through discussion with a third author (HC).

### Analysis

Qualitative content analysis was conducted for comprehensive description and interpretation. A qualitative data-based convergent synthesis design was adopted (28). We used Kuckartz' (29) guidance for conducting qualitative content analyses, using the same synthesis method for both qualitative and quantitative studies. Thus, quantitative data were transformed into qualitative categories in order to explore similarities and/or differences between studies. The quantitative data were also examined in terms of their stigma results, and narratively described results were coded. Any evidence found was summarized correspondingly. We used the software MAXQDA (Version 2018) to manage the storage of analysis. Extracted results were read several times, and memos were made to become immersed in the meaning. Sub-categories were compiled inductively and material-driven. Out of the formulated sub-categories, generic categories were abstracted, describing themes that emerged. Those generic categories were grouped into main categories with the help of comprehensive stigma frameworks (18, 19). The complete data (i.e., extracted results of interest) was coded with those main categories.^2^,^2^ Coding was done independently by two researchers (L-MD, FS) to ensure the reliability of data analysis. Discrepancies were resolved through discussion with HC.

## Results

### Search Outcome

**Figure 2:**
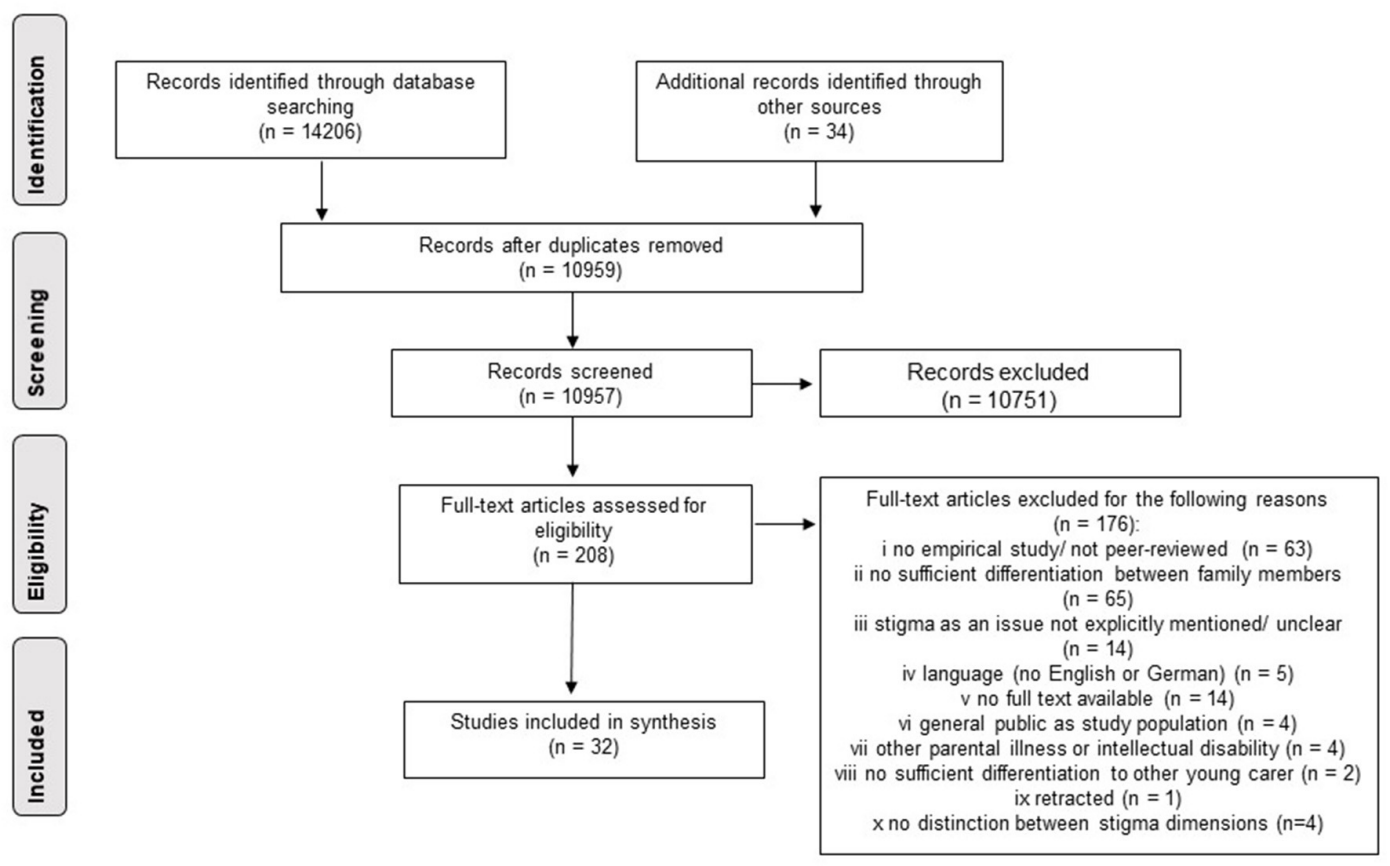
PRISMA flow chart demonstrating study selection.

Most studies were excluded at the title/abstract screening stage (see Figure 2), leaving 208 for full-text screening. Most of the studies were excluded at this stage because we could not differentiate their findings to other family members well enough, or because studies were not empirical or not peer-reviewed. A few quantitative studies also had to be excluded in the last stage because they measured unclear and mixed-up stigma dimensions, making it impossible to assign them to individual dimensions (see Figure 1). We were ultimately able to include a total of 32 articles from 30 studies^3^.

### Study and Population Characteristics

The majority of studies (30 ≜ 93.8 %) were qualitative; only one quantitative and one mixed method study were included, Table 1. Most of the studies were conducted in Europe (20 ≜ 62.5 %). Half of them had minor children as participants, the other half had adult children; one study combined the two. We ended up including more than half of the studies that had not aimed to explore stigma in this population, but aimed to capture their (everyday) experiences and burden. Stigma then emerged as one of the central themes in these studies. However, this also meant that most of the studies lacked a clear definition of stigma. Supplementary Table S1 provides a detailed description of the included studies.

**Table 1:** Study characteristics.

**Characteristics**	***n* (% of 32 articles)**
**Publication date**	
2013–2019	21 (65.6)
2007–2012	7 (21.9)
2001–2006	4 (12.5)
**Country of origin**	
Europe	20 (62.5)
North America	5 (15.6)
Australia	4 (12.5)
Asia	2 (6.3)
Africa	1 (3.1)
**Study design**	
Qualitative	30 (93.8)
Quantitative	1 (3.1)
Mixed Methods	1 (3.1)
**Study population**	
COPMI	18 (56.2)
Children, including COPMI	2 (6.3)
Relatives, including COPMI	6 (18.7)
Children, parents and professionals	4 (12.5)
Young carer, including COPMI	2 (6.3)
**Age of COPMI**	
Aged above 18/21	14 (43.8)
6–22 years	14 (43.8)
Not reported /applicable	3 (9.3)
Both	1 (3.1)
**Parental mental illness**	
Diverse Disorders combined	11 (34.4)
Mental illness, not specified	7 (21.8)
Affective Disorder	4 (12.5)
Schizophrenia	4 (12.5)
Alcohol or Drug Dependence	4 (12.5)
Obsessive Compulsive Disorder (OCD)	2 (6.3)

### Quality Appraisal

The quality of the studies was very heterogeneous and overall, 13 of the included studies met 60% or more criteria (for details see Table 2). They ranged from zero to five in their score, meaning that they ranged from 0 to 100 % of the criteria met. In qualitative studies, that were assessed as being of lower quality, often a clear rationale regarding the data collection method, the method of analysis or interview guidelines was missing, or the study population was not sufficiently defined. Quantitative studies of lower quality did not include a representative sample for their target population and had a low risk of non-response bias. The mixed-method study especially lacked in contrasting the results from the two study approaches and bringing the results together.

**Table 2:** Quality assessment (MMAT).

**Criterion**	**Clear research questions?**	**Data address research questions?**	**Qualitative approach appropriate?**	**Data collection methods adequate?**	**Findings adequately derived?**	**Interpretation of results sufficiently substantiated?**	**Coherence between qualitative data sources, collection, analysis and interpretation**	**Overall quality of the study^a^**
**QUALITATIVE STUDIES**								
Blakeman et al. (33)	Yes	Can't tell	Yes	Can't tell	Yes	Yes	Can't tell	^***^/80%
Bolas et al. (34)	Yes	Yes	Yes	Can't tell	Can't tell	Can't tell	Can't tell	^*^/20%
Carroll and Tuason (35)	Yes	Yes	Yes	Yes	Yes	Can't tell	Yes	^****^/80%
Cogan et al. 2005 (31)	Yes	Yes	Yes	Can't tell	Yes	Yes	Yes	^****^/80%
Dam et al. 2018 (36)	Yes	Can't tell	Can't tell	Yes	Can't tell	Yes	Can't tell	^**^/40%
Davison and Scott (37)	Can't tell	Can't tell	Yes	Yes	Can't tell	Can't tell	Can't tell	^*^/20%
Haug Fjone et al. (38)	Yes	Yes	Yes	Yes	Can't tell	No	Can't tell	^**^/40%
Fudge and Mason (39)	Can't tell	Yes	Yes	Can't tell	Can't tell	No	Can't tell	^*^/20%
Griffiths et al. (40)	Yes	Yes	Can't tell	Can't tell	Can't tell	Yes	Yes	^**^/40%
Kadish (41)	Can't tell	Can't tell	Yes	Can't tell	Can't tell	Yes	Can't tell	^**^/40%
Karnieli-Miller et al. (42)	Yes	Yes	Yes	Can't tell	Yes	Yes	Yes	^****^/ 80%
Krupchanka et al. (43)	Yes	Yes	Yes	Yes	Yes	Yes	Yes	^*****^/ 100%
Leahy (44)	Yes	Can't tell	Yes	Can't tell	Can't tell	Can't tell	Can't tell	^*^/ 20%
Leinonen et al. (45)	Yes	Yes	Yes	Yes	Yes	Can't tell	Yes	^****^/ 80%
McCormack et al. (46)	Yes	Yes	Yes	Yes	Yes	Yes	Yes	^*****^/ 100%
Moore et al. (47)	Yes	Yes	Yes	Can't tell	Can't tell	Can't tell	Yes	^**^/ 40%
Mordoch and Hall (48)	Yes	Yes	Yes	Yes	Yes	Yes	Yes	^*****^/ 100%
Murphy et al. (49)	Yes	Can't tell	Yes	Yes	No	Yes	Can't tell	^***^/ 60%
Nieto-Rucian and Furness (50)	Yes	Yes	Yes	Yes	Can't tell	Can't tell	Yes	^***^/ 60%
Oskouie et al. (51)	Can't tell	Can't tell	Yes	No	Yes	Can't tell	Can't tell	^*^/20%
Östman (52)	Yes	Yes	Yes	Can't tell	No	No	No	^*^/ 20%
Rezayat et al. (53)	Yes	Yes	Yes	Can't tell	Yes	Yes	Yes	^****^/80%
Stengler-Wenzke et al. (54)	Yes	Yes	Yes	Can't tell	Yes	Yes	Yes	^****^/ 80%
Tabak et al. (55)	Yes	Can't tell	Can't tell	Can't tell	Can't tell	Yes	Yes	^**^/ 40%
Tamutiene and Jogaite (57)	Yes	Yes	Yes	Can't tell	Yes	Can't tell	Can't tell	^**^/ 40%
Trondsen and Tjora (58)	Yes	Yes	Yes	Yes	Can't tell	Yes	Yes	^****^/ 80%
van der Sanden et al. (22)	Yes	Yes	Can't tell	Yes	Yes	Can't tell	No	^**^/40%
van der Sanden et al. (30)	Yes	Yes	Yes	Can't tell	Yes	Yes	Yes	^****^/80%
Wahl et al. (59)	Can't tell	Yes	Can't tell	Can't tell	Can't tell	No	No	-/0%
Widemalm and Hjärthag (60)	Yes	Yes	Can't tell	Yes	Can't tell	Can't tell	Yes	^**^/40%
**Criterion**	**Clear research questions?**	**Data adress research questions?**	**Sampling strategy** **relevant to address research question?**	**Sample representative of the target population?**	**Measurements appropriate**?	**Risk of nonresponse bias low?**	**Statistical analysis appropriate to answer the research question?**	**Quality**
**QUANTITATIVE STUDIES**								
Haverfield and Theiss (61)	Yes	Yes	Yes	No	Yes	No	Can't tell	^**^/40%
**MIXED METHODS STUDY**								
Cogan et al. (32)	Yes	Can't tell	Can't tell	Can't tell	Can't tell	No	Can't tell	-/0%

a *The quality score was calculated according to the official advices of the authors of the MMAT tool (http://mixedmethodsappraisaltoolpublic.pbworks.com/w/file/fetch/140056890/Reporting%20the%20results%20of%20the%20MMAT.pdf).*

* *20% of the criteria met;*

** *40% of the criteria met;*

*** *60% of the criteria met;*

**** *80% of the criteria met;*

***** *100% of the criteria met*.

### Identified Aspects of Stigma Related to Parental Mental Illness

Following our analysis, we identified four different stigma dimensions described by or regarding children that are well-known from the stigma experiences of people who have a stigmatized condition themselves: *experienced SBA, anticipated SBA, affiliate (internalized) stigma*, as well as *structural discrimination*. Within these main categories, we identified various subcategories that are described in the following sections. Table 3 reports the category system for stigma dimensions, their frequencies, and associated studies.

**Table 3:** Identified aspects of stigma related to parental mental illness.

**Main category**	**Generic category**	**Sub-category**	**References**
**Experienced SBA (29)**	Having unmet emotional needs (18)^a^	•Experiencing withdrawal and rejection (6) •Experiencing others who cannot understand or cope with parental mental illness (7)	• (33, 36, 57, 62, 63) • (35, 39, 48–50)
		•Being confronted with inappropriate language and statements about mental illness (5)	• (30, 42, 47, 57)
	Experiencing hostile behaviours of others (11)	•Being the victim of bullying and laughter (8)	• (36, 39, 45, 48, 62)
		•Being confronted with hurtful words (3)	• (36, 51, 59)
**Anticipated SBA (29)**	Fearing hostile behaviors of others (6)	•Fearing gossip (2) •Fearing ridicule (4)	• (33, 48) • (32, 33, 49, 57)
	Fearing of negative attitudes and ascriptions (15)	•Fear of other people's negative attitudes (5)	• (33, 40, 52, 59)
		•Fear of being labeled as “different“ (7) •Fear for parents to be described as bad (3)	• (30, 46, 48, 50, 52, 60, 64) • (39, 57)
	Fearing others' lack of understanding and rejection (8)	•Fearing others' lack of knowledge and understanding (4)	• (37, 57, 59, 62)
		•Fearing withdrawal and rejection (4)	• (22, 33, 34, 41)
**Affiliate stigma (51)**	Perceiving themselves as being contaminated (9)	•Struggling to avoid being contaminated (8)	• (30, 35–37, 54, 58, 64)
		•Fearful of passing it on (1)	• (30)
	Perceiving themselves as being inferior (42)	•Feeling ashamed and embarrassed (30)	• (22, 30–36, 40, 41, 44, 46, 49–51, 54, 55, 57–61, 64)
		•Perceiving themselves as different from others (9)	• (30, 33, 41, 46, 49, 50, 58, 64)
		•Self-blaming (3)	• (31, 64)
**Structural discrimination (35)**	Perceiving discrimination within the mental health system (10)	•Not receiving information in hospital (3) •Experiencing cold furniture and atmosphere in the hospital (1)	• (32, 36) • (36)
		•Experiencing insensitive treatment by professionals (3)	• (36, 50)
		•Perceiving a lack of care provided for the parent (3)	• (43, 60)
	Perceiving discrimination within the education system (10)	•Needing more education in school about mental illness (1)	• (32)
		•Feeling teachers ignore their parents' mental illness (7)	• (33, 36, 57, 62)
		•Disadvantages (2)	• (39, 62)
	Perceiving a lack of knowledge provided/societal taboo (8)	•Needing more information and openness from society (8)	• (36, 37, 39, 49, 50, 58)
	Perceiving discrimination within the police (2)	•Experiencing discrimination during contacts with the people (1)	• (43)
		•Feeling ignored by the police (1)	• (57)
	Perceiving discrimination within media (2)	•Perceiving media representations of people with mental illness as bad (2)	• (32)
	Perceiving discrimination within social work (3)	•Feeling unseen by social workers (1) •Perceiving social workers as a source of control rather than help (2)	• (57) • (57)

a *Numbers in brackets show how many codes we identified for those categories*.

#### Experienced SBA—Having Experienced Unpleasant (Re)Actions of Others

We identified two generic categories of SBA in this section: (1) having unmet emotional needs, and (2) experiencing the hostile behaviors of others. Both revealed various subcategories and are now described in more detail.

##### Having Unmet Emotional Needs

###### Being Confronted With Inappropriate Language and Contents About Mental Illness.

Some children reported that they were unhappy about being classified in a way that they would not describe themselves, e.g., being called a “young carer”, and were unhappy about not being asked whether they identified with that designation (47). Some participants reported that others gave them unwanted advice, such as recommending them to use birth control to avoid passing on mental illness to their children (42). One child remembers that her boyfriend's family reacted to her parent's mental illness saying “*What if you two got married and you have children and they have a mental illness?*” (42).

###### Experiencing Others Who Cannot Understand Them or Cope With Their Situation.

Often, children reported that people did not know how to act and cope with the information of parental illness [e.g., (48)]. One participant stated that they wished there was greater community awareness so “*that they know there's nothing wrong—anyone could get it if they get stressed out—they could get it*” (39). Some also reported that others failed to intervene, could make things worse (35), or that friends did not understand their experiences and they were disappointed in how friends responded (48). Some children emphasized that they wanted to talk to family members, but their parents and grandparents refused, and they felt unable to talk openly about such family issues (49, 50). A few children described having nobody to talk to and ask for help. They believed that this was due to taboos and others' ignorant behaviors [e.g., (35, 36)], having direct influences on their social interactions.

###### Experiencing Withdrawal and Rejection.

In addition, children perceived that others actively avoided them: for example, an adult child in Dam's study described, respectively (36) “*When I met someone, they ignored me; they went over to the other side of the road”*, as a result of having lived in a small community with a mentally-ill parent. Moreover, participants reported family members breaking off their relationship with them after finding out about their parent's illness (65), or experiencing the loss of friendships: “*...my friend's mother called me over and told me that I couldn't be friends with her, because I'll be the same as my mother”* (57).

##### Experiences of Hostile Behaviors of Others

###### Being the Victim of Bullying and Laughter.

Bullying (e.g., “*I was bullied, the others laughed at me*.”) (36) as one form of overt hostile behavior, was frequently mentioned: Children reported being: teased, laughed at, drawn into fights, and treated like a “*leper*” (36). A participant in Mordoch and Hall's (48) study stated that they “*Sometimes [I] get in fights by accident”* because other people labeled them as different. In another study, one participant wished that others would “*stop teasing*” (39).

###### Being Confronted With Hurtful Words.

Furthermore, children reported that their peers used stigmatizing words when talking about their ill parents, like ‘crazy' (36), ‘mad' (51), or their living situation as a ‘crazy house' (59).

#### Anticipated SBA—Fearing Reactions of Others in the Future

Regarding anticipated stigma in the future, similar topics that we identified in the experienced SBA emerged for anticipated SBA: Children feared overt hostile behaviors from others, as well as negative attitudes and ascriptions. They were also worried that others would not be able to understand and would reject them. Those generic categories are now presented together with their sub-categories.

##### Fearing Hostile Behaviors of Others

###### Fearing Ridicule.

When children were asked why they hesitate to tell others about their parent's mental illness, they described being afraid others would laugh at them (32, 57), and being afraid of being teased or bullied (33, 49).

###### Fearing Gossip.

Furthermore they feared that other people would share this information and gossip about them (33, 48). One participant describes her tendency to isolate herself due to fearing gossip and accompanying discrimination: “*I was not able to develop close friendships at school because I had this fear of them coming home with me and seeing what it was like and then telling everybody at school and I would be a laughing stock”* (33).

##### Fear of Negative Attitudes and Ascriptions

###### Fear of Being Labeled as “Different”.

Children frequently feared being labeled as “*different from others”* if people discovered their parent's mental illness. They thought people might believe their mothers, for example, might not act like other mothers [e.g., (60, 64)], or that they are “*berserk*” (33).

###### Fear of Other People's Negative Attitudes.

They also feared that others might be scared or have *negative attitudes* toward them or their family (52). One participant stated that they do not invite friends home because they are scared that others might think something bad about their family (33). Another child reported being scared of being judged or criticized for having a “*whacko*” father (46).

###### Fear for Parents to be Described as Bad.

Some children worried others might think their parents could not take care of them because of their mental illness and that they might be taken away (39, 57). One participant stated: “(…) *It's just that I'm scared they'll put it down to her being a bad mum... she's not a bad mum, she loves us*” (32).

##### Fearing Others' Lack of Understanding and Rejection

###### Fearing Others' Lack of Knowledge About Mental Illness and Lack of Understanding.

Moreover, children worried that others have little knowledge of mental illness (59) and that their situation and experiences will not be understood (57, 62). In some instances, they anticipated others' misconceptions and did not get any help, or tell anybody about it “*because some children just do not know what this [mental illness] is and then just tell people that it is something really bad, that is stupid for you”* (59). On the other hand, two participants in the study from Davison and Scott (37) anticipated that personal support interventions could even make things worse for children, in case other people try too hard to help them, leading to more worries and fear: “*People sort of trying to help them too much when they don't have a problem could make people overthink things… It might increase the fear factor…”*.

###### Fearing Withdrawal and Rejection.

They also feared others might withdraw from, reject (34, 41), or exclude them (33). Some children also feared losing contacts, such as relationships when their partner discovers their parent's mental illness: “*I've got a girlfriend, should I tell her… I've been going out with her for 3 or 4 months…but she's never met my mother. And I do have a particular reason, for, like, putting it off as long as possible. And that, oh I don't know, feeling embarrassed, it's just the idea, I don't know actually. Let her get to know me first and if she likes me enough, then it won't make any difference any more*” (22).

#### Affiliate Stigma—Internalizing the Stigma

The self-stigmatization of children manifested in two different ways, namely the two generic categories we identified: (1) perceiving themselves as being contaminated, and (2) perceiving themselves as being inferior. Both are now presented in more detail together with their subcategories.

##### Perceiving Themselves as Being Contaminated

###### Struggling to Avoid Being Contaminated.

van der Sanden et al. (30) found that adult children feared contamination. The fear of being contaminated relied on one of two components: Either (1) children knew about their status of being a member of an at-risk group for developing a mental illness, which could result in fear and self-reflexive sensitivity related to possible symptoms (58); or (2) they feared being seen as connected to their ill parent so closely that they would also be considered “*crazy*” (35): “*My Mom is crazy, so they are going to think I am crazy”. One* child reported that parental mental illness is something that cannot be discussed because it is like a “*an infected tumor never to heal*” (P9) (36). An online self-help group for adolescents with a mentally ill parent reduced these fears, as one participant described: “*They seemed quite normal; after all, it was just that they had a terrible situation. Then I thought, “Oh my God, they are never going to be mentally ill like that.”... I felt it was less likely that I would also become like my mom.... Because [one of the participants] had a boyfriend, I felt that I too had a chance to get a boyfriend, friends, and live a completely normal life […]”* (58).

###### Fearful of Passing It on.

One child reported fearing stigmatization being passed on to the next generation when they have children themselves (30), and thus characterize themselves as a possible source for transmitting mental illness.

##### Perceiving Themselves as Being Inferior

###### Feeling Ashamed and Embarrassed.

Beliefs of being inferior were mainly characterized by statements related to shame and embarrassment: Children often viewed their parents' mental illness as a “*secret*” (35, 44) that must be kept “*behind closed doors”* (31). One child described doing this to protect their parent from other people's stigmatizing behaviors (44). Often, children mentioned that they did not want anybody to know about their parent's mental health issues, but sometimes were unsure why they were ashamed, or did not want anybody to know: “*And I do have a particular reason, for, like, putting it off as long as possible. And that, oh I don't know, feeling embarrassed, it's just the idea, I don't know actually”* (22). Sometimes participants even reflected that this secrecy would keep them from establishing deep relationships, for example one participant said*: “I avoid entering deep relations with others due to fear of explaining my situation”* (51). One son of a father with a mental illness stated: “*I wish my father had another disease and didn't suffer from mental illness. Father's illness is very bad for me. I am ashamed to speak about it and to communicate with others”* (51). In the quantitative study by Haverfield and Theiss (61), intrafamilial topic avoidance regarding parental illness was also associated with magnified avoidance of disclosing the topic to others—this was evident in the male participants at least.

###### Perceiving Themselves as Different to Others.

Frequently, this shame, embarrassment, and disclosure was described as a result of being seen as “different”, “abnormal”, or “wrong”. A commonly mentioned experience for children was that they felt different from their peers (41, 49, 50), and the feeling of being different led them to think dichotomously, i.e., believing other families were “*right*”, while their own family was “*wrong”: “Our family was wrong…we were just a dysfunctional, broken family, we just weren't normal like other families”* (46).

###### Self-Blaming.

Self-blame was also identified in children: They were more likely to blame themselves for their parents' difficult situation than were children whose parents have no mental illness (31). One child explained: “*I sometimes think it's my fault, I blame myself for the way my dad is feeling, even though he tells me it's not”* (31).

#### Structural Discrimination—Perceiving Inequalities in Institutions and Within the Society

We identified three generic categories within the structural discrimination facet now discussed in more detail, namely perceived discrimination within (1) the mental health system; (2) the education system, and (3) other sources of discrimination

##### Perceiving Discrimination Within the Mental Health System.

Most of the references for structural discrimination related to interactions with the healthcare system: Children described interactions at the interpersonal level within the health care system, such as being treated insensitively, and perceived that doctors were afraid of them and maintained an emotional distance (50): “*To visit mum in the hospital was like coming to an office. It was a nonexistent relationship, you know. Hello, Goodbye, You must go in that direction (P4)”* (36). Furthermore, they felt ignored by staff (36). Due to feeling fear and insecure when visiting their parents in hospital, they wished that someone would stay with them and talk to them about their situation (36). On a broader institutional level, children strongly requested more general information about mental illness from mental health workers (32). Children remarked that the cold furniture in the hospitals made them feel uncomfortable (36). They also mention a general lack of care for their parents. They had the impression that their parents were getting inadequate healthcare, and were discharged due to inadequate bed capacity, leaving the children in charge of their parents: “*After 2 weeks she was sent home, even though she didn't feel ready. I and my sister had to move home and take care of her ourselves. I will never forgive the psychiatric institution for what they did to me and my sister. We literally had to act like ‘extra mothers' to our own mother”* (60).

##### Perceiving Discrimination Within the Education System

The education system was also identified as a source of structural discrimination: children complained of a lack of education about mental illness at school (32). They criticized a lack of understanding about students who have to look after their parents (39), with teachers ignoring their situation instead of talking with them “*Well it's a very stereotypical town where you would never want to admit that anything is wrong... so a lot of it was hush hush. So, like, we'll give you a little extension on your homework. If you need someone to talk to you can go and talk to somebody. But we're not going to like, raise any ags*” (33); see also (36, 62).

##### Other Sources of Structural Discrimination

Other sources of structural discrimination were the police (43, 57), media (31), and social work (57). Events experienced there or unclarified mandates from the institutions led to a mistrust in the children, who felt abandoned or did not seek help for fear of losing their parents (57). Above all, the general taboo around this topic was identified, which is not inherent in a specific organization, but rather within society in general, with people not talking to children about these problems, which might hinder children obtaining appropriate and useful information (49, 50, 58). Not getting any information about parental mental illness and its heritability or about their risks in general left children to overestimate their risk of developing a bipolar disorder themselves (37). One participant said: “*I think knowing about the risk would be really helpful—I've always worried that I would get it (BD)”* (37).

## Discussion

We identified 32 studies for this review that investigated or addressed the stigma experiences of children with parents with a mental illness. The studies are heterogeneous in their design and methodological quality. Low-quality papers failed to state a comprehensible rationale for their data collection or analysis, or failed to sufficiently describe (or even recruit) a representative sample. More than half of the studies did not investigate stigma primarily, rather, they only identified stigma as an important problem for this population. Due to a mixed-up and vague measurement of stigma dimensions, we were only able to include one quantitative study and one mixed-method study. Qualitative content analysis was done inductively, and we were able to cluster our results in four stigma dimensions that match those absorbed in stigma theories (18, 19, 66): experienced SBA, anticipated SBA, affiliate stigma, and structural discrimination. We were able to fill those dimensions with content that is specific for the stigma experiences that the children of parents with a mental illness suffer, and we have thus closed the gap in showing that these theoretical categories were confirmed with qualitative content analyses, and that the stigma experiences that the children expressed align with theory. The affiliate stigma dimensions we identified highlight the close connections between stigma and feelings of guilt, isolation, and secrecy in affected children, supporting results of the review by Reupert et al. (25).

While this review does not enable causal conclusions about the health risks after stigmatizing experiences, our results demonstrate that such experiences are indeed stressful and likely to affect the health and well-being of those stigmatized, as conceptualized in the TTMD model (1, 7). Stigma is therefore no mere isolated mechanism that impairs a child's well-being—it is a mechanism interwoven and manifold on different levels in families with mental illness.

### Dimensions of Stigma

#### Experienced SBA

Our findings regarding *experienced SBA* reveal that children do have experiences that are very specific to their role as the offspring of a parent with a mental illness. At school and in other areas of social life, they are bullied and teased. They often feel incapable of making strong connections with other people in a satisfying way, as they often sense the need to keep their parent's mental illness a secret. In their early years, children can experience—depending on the severity and symptoms of their parent's mental illness and the existence of reliable other family members—a lack of emotional support within their family (55, 56). For them therefore, relationships functioning as a secure base outside the family might be even more important than for other children. In adolescence, this can be problematic, as it is a life phase characterized by various developmental steps that include the establishment of new and meaningful relationships outside the primary family, and developing a strong sense of identity (70).

#### Anticipated SBA

As far as we know, *anticipated stigma* has not been described in the literature to characterize family members of people with a mental illness. This SBA dimension was identified in this review and was closely connected to feelings of fear and worry. This could be an important aspect of hiding the parent's mental illness and failing to seek help, and it supports the evidence from studies on the primary recipients of mental illness stigma, which showed that anticipated stigma and discrimination are key reasons for not seeking help [e.g.,(67)].

#### Affiliate Stigma

This review sheds light on how children might *internalize the stigma*. While some quantitative studies claimed that children are more apt to experience contamination, and that blame and shame play more important roles for the parents and spouses of people with a mental illness (13, 68), our review's results show that blame and shame are also important for children. This is especially true regarding the negative perception of themselves as being different from others—thereby perceiving themselves as inferior. This review reveals this as a specific and frequently mentioned self-stigma for children. This finding is in line with general stigma theories for primary recipients, which posit that a core component of stigma is the mark of otherness, which is then followed by negative evaluation (69). Children seem to internalize this *otherness* by feeling a sense of being “abnormal” or “wrong”. Park and Park's (71) family-stigma concepts could explain the frequent mention of feeling “different” identified in this review. According to their theory, general family stigma arises for two reasons: (1) it might be due to negative events (like the parental illness) or (2) to exceptional family structures. As structures in families with a parental illness differ in many ways [e.g., (53)] from those of other families, we can assume that such children are perceived and perceive themselves differently because of both labels. This may provide an explanation for the perceived family otherness so frequently cited in this review. Often, this otherness was associated with inferiority, which was considered a reason that this must be kept a secret and possibly keeping children and their families from seeking help. As previous research has shown for relatives of people with a mental illness, internalized stigmatization is especially associated with psychological problems such as reduced self-esteem—causing both psychological distress and lower quality of life (72, 73).

#### Structural Discrimination

Furthermore, our review demonstrates that *structural discrimination* is a key stigma dimension not only for those suffering from mental illness, but also for their children. A recently published systematic review (25) described structural discrimination being detected within law, medicine and education: in legal terms, for example, the greater likelihood of parents losing custody of their child, as well as being accused of having an “unsound mind” in all matters for people with mental illness. These are undoubtedly important findings for families and especially parents with a mental illness that probably affect the entire family system including children. Nevertheless, our review suggests that there is structural discrimination surpassing those descriptions that is described by the children themselves and which we clustered as an element of structural discrimination, as it occurs within institutions or characterizes a societal attitude that differs from individual attitudes. The results of the current review show that there is an inherent stigmatizing structure in different areas of society, e.g., schools and hospitals. For children, structural discrimination includes their parents' experiences, which in turn have consequences for themselves. This form of stigma also entails disadvantages personally experienced through various institutions. There is evidence that healthcare systems are a main source of children's structural discrimination. Children are affected by the lack of care offered or provided to their parents: They are often put in charge of taking care of their parents when hospitals/adult mental health professionals turn them away. Generally, the most significant themes within structural discrimination is the lack of education and information about mental illness, and society's tendency to ignore the subject. This prevents children from getting the necessary information and support to establish a healthy way of coping with their situation, like talking to others. Mental health literacy and positive family communication about the disorder are both known to be major protective factors for children (74, 75). However, the results of this review reveal a societal structure in which they do not get enough information at all.

### Strengths and Limitations

This is the first systematic mixed-method review to explore and collate different facets of stigma for children of parents with a mental illness, focusing on the experience level of children themselves and grouping those facets into dimensions to inform how we conceive of affiliate stigma on the experience level. Having included both qualitative and quantitative study designs is a strength of this review—the topic is thus covered from different perspectives and with various measures. Furthermore, including both under-age and adult children deepens our knowledge of their stigma experiences: Views from children still living with their parent (as in about half of the included studies) provide information on how they are assessing their current situation. Retrospective views from adult children add important information, especially as some thoughts cannot be expressed and understood by children of younger ages, as they might not be aware of stigmatizing structures. For instance, children cannot anticipate that some stigmatizing experiences might influence their future in a specific way.

However, retrospective views can also lead to bias. Memories can change, and ascriptions made more easily [recall-bias (76)]. Secondly, we only included studies written in English or German, as well as peer-reviewed publications, and no gray literature. Thus, it is possible some important literature is missing. In addition, the studies mostly came from Western countries, restricting our results and conclusions to those cultures. As stigma is the result of the social context and therefore differs over time and cultures, the present review provides an overview of possible topics relevant for children, but to ensure generalizability, a wider range of cultures would need to be addressed. As most of the studies came from Europe, this review mainly contributes to understanding how stigma affects children of parents with a mental illness in European countries, which might well differ in other countries. Third, the quality of studies we included ranged from very low to very high quality. In many qualitative studies, data collection methods, analyses methods, as well as interview guidelines were not specified, which made it difficult to assess their quality and adequacy. In addition, we mainly included qualitative studies as we could only include one quantitative and one mixed-method study. The other quantitative studies of potential relevance had to be excluded because of their low relevance for this specific population, i.e., they only assessed which kinship status experienced more or less stigma by association, or they mixed up different stigma measures which made it impossible to classify the stigma.

### Implications for Policy, Practice, and Future Research Directions

We propose several policy, practice, and research implications as a result of our review. Our findings highlight that much more education about mental illnesses for the general public, and especially children, is essential. Enhanced education about the risks and potential support networks that can lower those risks could lead to addressing some of these misconceptions and fears children may have: It could pave the way for a realistic evaluation of their own risks, but also various means of dealing with these risks. According to the literature on the manifold effects of stigma on relatives of people with mental illness [e.g., (14, 16, 22)] and our study results, we can assume that stigma is a major factor contributing to the transgenerational transmission of mental disorders. Thus, at the level of social factors contributing to mental illness, stigma represents a target for prevention and intervention programs for the children of parents with a mental illness. This should be highlighted when considering stigma for this population, and could make an important difference in developing strategies to reduce stigma. Overall, stigma-reducing interventions, in terms of minimizing the public stigma of people with a mental illness, as well as encouraging more openness and acceptance by “normalizing” mental health problems, and empowering people to talk openly about mental illness, would be a further promising step to escape the vicious circle of mental illness stigma that contributes to the transgenerational transmission of mental illness.

The review has shown that adult health care services seem to be an important source of discrimination: It is not just the fact that children feel so ignored and insensitively treated by healthcare staff, but also the atmospheres that keep kids from feeling comfortable that should be addressed. Hospital and psychiatric staff for adult health care should undergo training in engaging with the children of parents with a mental illness [see also (77)]. Other support structures need to be established to lessen the pressure children feel that it is up to them to look after their parents.

Our findings emphasize the need for individual investigations to determine whether different aspects are important for specific family members. They might indeed differ more than previous research suggests, considering the homogeneity of the questionnaires used for all family members. As very few studies have provided evidence about which aspects influence how strongly children experience stigma, there is a need for researchers to investigate additional factors in more detail and across more types of parental mental illnesses. Future research should focus on a deeper differentiation of stigma dimensions and how they interact, and on their impact on the health and well-being of this specific population, which is worldwide, large, and carries such a high risk.

## Data Availability Statement

The original contributions presented in the study are included in the article/Supplementary Material, further inquiries can be directed to the corresponding author/s.

## Author Contributions

L-MD designed and completed the study, wrote the protocol, was involved as the main person in charge in all analyses steps, and wrote the manuscript. JF did the independent full text screening as the second rater and was involved in the studies' inclusion process. DP did the data extraction as second rater and was involved in the studies' inclusion process. JLP did the quality assessment for qualitative studies as a second rater, supported the intersubjective validity by discussing the categories, and supported the writing of the manuscript by reviewing and editing. AB did the quality assessment for quantitative studies and the mixed method study as a second rater, supported the intersubjective validity by discussing the categories, and supported the writing of the manuscript by reviewing and editing. HC functions as PhD advisor and supervised the study and preparation of manuscript and supported finding agreement between two independent raters by discussing ambiguous study inclusions. All authors contributed to the article and approved the submitted version.

## Funding

The paper was written as part of a 4-year research project the Village funded by the Austrian Federal Ministry of Health—Science and Research through the Open Innovation in Science Center at the Ludwig Boltzmann Gesellschaft GmbH, hosted at the Medical University of Innsbruck, with collaboration of Co-Investigator institutions. The funders did not influence the collection, analysis, or interpretation of data, and played no role in writing the manuscript.

## Conflict of Interest

The authors declare that the research was conducted in the absence of any commercial or financial relationships that could be construed as a potential conflict of interest.

## Publisher's Note

All claims expressed in this article are solely those of the authors and do not necessarily represent those of their affiliated organizations, or those of the publisher, the editors and the reviewers. Any product that may be evaluated in this article, or claim that may be made by its manufacturer, is not guaranteed or endorsed by the publisher.

## Footnotes

^1^Throughout our paper we mainly use the term “children” when referring to the children of people with a mental illness as this is the population of interest. This can also mean that we are talking about the adult children of parents with a mental illness—depending on the study population in the original papers—by using the term ‘children', referring to children of parents with a mental illness.

^2^The coding guideline is available upon request.

^3^Van der Sanden (22) and van der Sanden (30) stem from one study with different research questions but the same data; Cogan et al. (31) and Cogan et al. (32) also stem from one study.
