# Supplementary material for: How Do Children of Parents With Mental Illness Experience Stigma? A Systematic Mixed Studies Review
Source: Front Psychiatry. 2022 Feb 18;13:813519. doi: 10.3389/fpsyt.2022.813519 (PMC8894251; doi:10.3389/fpsyt.2022.813519)
Supplement: Supplementary file 1 [file Table_1.docx]

**Supplementary material**

**Table S1.** Study details of included studies

| **No.** | **Author, year,**  **country of origin** | **General study objective** | **Data collection (Data analysis) method** | **Study population** | **Sample size (n)** | **Age**  **(COPMI)** | **Parental mental illness** | **Stigma domains found in study findings** |
| --- | --- | --- | --- | --- | --- | --- | --- | --- |
| *Qualitative studies* | |  |  |  |  |  |  |  |
| 1. | Blakeman et al. 2019  UK | Exploring the experiences made as a child and as an adult, and the ways they shape their adult lives | Interviews | Adult children who as a child, lived with, or had regular contact with, a parent with psychosis | 5 | 19-58 | Psychosis | Received Stigma; Anticipated Stigma: Affiliate Stigma; Structural Discrimination |
| 2. | (Bolas et al. 2007)  UK | Exploring psychological processes that determine & maintain relationship between young carer’s role and their well-being. | Semi-structured interviews (IPA) | Young carer | 5 | Participants: 14-18, the only COPMI: 16 | The only COPMI in this study: long-term alcoholism, resulting in nervous system damage | Affiliate Stigma  Anticipated stigma |
| 3. | (Carroll und Tuason 2015)  USA | Stigma experiences and associations with the processes of developing & maintaining a same-sex sexual identity. | Semi-structured interviews (CQR) | Lesbian daughters of mothers with SMI | 12 | 26-76 (M=42.30, SD=12.48) | Bipolar disorder (6), major depressive disorder with psychotic features (3), borderline personality disorder (2); OCD (1); partially with comorbid disorders | Received stigma;  Affiliate stigma |
| 4. | (Cogan et al. 2005b) UK | Comparing the understanding of mental health problems. | semi-structured interview (interactive model) | Children affected by parental mental health problems, and children of well parents | 40 (20 COPMI,  20 non-COPMI) | 12-17 (M=13.81, SD=1.74) | ICD-10 diagnosis of an affective illness | Anticipated stigma; Affiliate stigma; Structural discrimination |
| 5. | (Dam et al. 2018)  Faroe Islands, Denmark | Exploring recalled childhood life of adult COPMI. | Semi-structured interviews (content analysis) | Adults, who as children lived with a parent experiencing mental illness | 11 | 18-49 | Bipolar disorder (5), schizophrenia (6) | Received stigma;  Affiliate stigma;  Structural discrimination |
| 6. | Davison & Scott, 2016, UK | To examine  similarities and differences between children and parents in their attitudes  toward BD and to interventions for individuals who are asymptomatic  but at above average risk of developing BD in the future. | Interviews (qualitative thematic analysis) | Children of parents with bipolar disorder (BD) and parents with BD | 7 children; and 6 unrelated parents | Children had to be 12-26 to be eligible for participation *(no data provided for the actual sample)* | Bipolar disorder | Anticipated stigma, Affiliate stigma, Structural discrimination |
| 7. | (Haug Fjone et al. 2009)  Norway | Examining strategies to avoid stigma & self-stigmatization and to be viewed as ‘normal’. | In-depth interviews (Meaning condensation and categorization) | Children with a parent or both parents suffering from mental health distress during their childhood | 20 | 8-22 | Not further specified | Anticipated stigma;  Affiliate Stigma |
| 8. | (Fudge und Mason 2004)  Australia | Gaining feedback on key areas of a discussion paper. | Focus groups and peer interviews (no analysis method explicitly mentioned) | COPMI | 58 (33 children,  25 young people) | Children: 7-12, young people: 13-20 | Not further specified | Received stigma;  Anticipated stigma;  Structural discrimination |
| 9. | (Griffiths et al. 2012)  UK | Exploring experiences of COPMI | Semi-structured interviews (inductive thematic analysis) | COPMI | 10 | 13-19 | OCD | Anticipated stigma;  Affiliate stigma |
| 10. | (Kadish 2015)  South Africa | Ascertaining the believed impact on psychological development. | Semi-structured interviews (thematic content analysis) | Women who were raised by mothers suffering psychosis | 5 | 28-53 | Bipolar I disorder, schizoaffective disorder, and schizophrenia | Anticipated stigma;  Affiliate stigma |
| 11. | (Karnieli-Miller et al. 2013)  USA | Exploring experiences and efforts to cope with mental illness stigma in social encounters. | Focus groups (qualitative immersion/crystallization analysis) | Family members of people with MI | 14 relatives (2 adult COPMI) | 30.5+-16.4 (2 adult COPMI) | Schizophrenia or bipolar disorder | Received stigma |
| 12. | (Krupchanka et al. 2016)  Belarus | Investigating the experience of stigma in Belarus. | semi-structured in-depth interviews (thematic analysis) | Relatives | 20 | Median age = 52; subgroup ages N.A. | Schizophrenia | Structural discrimination |
| 13. | (Leahy 2015)  USA | Exploring reflections on their school experiences and understand the effects of childhood trauma. | Biographical interviews (Grounded Theory) | Adult children who grew up during their school days with a mentally ill parent. | 8 | 30-64 | Severe mental illness, including paranoid schizophrenia (3), the others not further specified | Affiliate stigma |
| 14. | (Leinonen et al. 2016)  Finland | Examining the arising and negotiation of stigma of a parental mental disorder. | Single-case study through interviews (Dialogical analysis) | Child of parent with mental illness | 1 | 8 | Not further specified | Received stigma |
| 15. | (McCormack et al. 2016)  Australia | Investigating subjective interpretations of the ‘lived’ experiences. | Semi-structured interviews (IPA) | Adult children who as children lived (at least in part) with biological parent mental ill-health | 7 | 20-45 | Major depressive disorders (4), alcohol dependence (2), schizo-affective disorder (1) | Anticipated stigma;  Affiliate stigma |
| 16. | (Moore et al. 2010)  Australia | Exploring and comparing specific needs. | Interviews & focus groups (‘meaning focused’ analysis approach) | Young people caring for a parent | 15 | 11-17 | Alcohol or other drug issue | Received stigma |
| 17. | (Mordoch und Hall 2008)  Canada | Examining how children managed their experiences. | demographic questionnaires, semi-structured interviews, observation, drawing (grounded theory) | Children living with a parent who has MI | 22 (from  14 families) | 6-16 | Depression, schizophrenia or bipolar illness | Received stigma;  Anticipated stigma |
| 18. | (Murphy et al. 2015)  Australia | Investigating experiences of childhood parental mental illness. | Narrative enquiry (narrative analysis) | Adult COPMI | 13 | 30-78 | Participants self-reported diagnosis: schizophrenia, psychosis, depression or other mood disorder | Received stigma;  Anticipated stigma;  Affiliate stigma;  Structural discrimination |
| 19. | (Nieto-Rucian und Furness 2017)  Spain | Exploring perceptions of the effects of experiences upon their development and their impact upon their adult lives. | In-depth interviews (IPA) | Adult children who grew up with a parent with schizophrenia | 6 | 28-37 (average age 33) | Schizophrenia | Received stigma;  Anticipated stigma;  Affiliate stigma;  Structural discrimination |
| 20. | Oskouie et al., 2011, Iran | to explore and describe  the outcomes of parents’ mental  illness on their children | Semi-structured interviews (Grounded Theory) | COPMI, their families, and health care practitioners | 17 (10 children) | 17-26 | Not specified | Affiliate stigma; Experienced stigma |
| 21. | (Ostman 2008)  Sweden | Investigating experiences of their life situation. | Interviews (inductive thematic analysis) | COPMI | 8 | 10-18 | Schizophrenia (4), affective disorder (4) | Anticipated stigma |
| 22. | Rezayat et al., 2019  Iran | Exploring the process of addressing stigma in people with mental illness. | Combination of unstructured and semi- -structured interviews | Patients and family members | 7 patients and 9 family members ( 1 COPMI) | 22-58 years (mean age = 46 years) the only COPMI: 22 years | schizophrenia spectrum  disorders | Received stigma |
| 23. | (Stengler-Wenzke et al. 2004)  Germany | Describing experiences of stigmatization and discrimination in everyday lives. | Narrative interviews (grounded theory and thematic field analysis) | Family members of patients with obsessive-compulsive disorder | 22  (3 of  them COPMI) | COPMI between 19-38 years | OCD | Affiliate stigma |
| 24. | (Tabak et al. 2016)  England, Finland, Germany, Italy, Norway, Poland and Scotland | Analyzing needs, expectations and consequences for children living with a parent with MI. | Focus groups & interviews (framework analysis) | Professionals, parents with mental illness, adult children and partners of parents with mental illness | 96 | / | / | Affiliate stigma |
| 25. | (Tamutienė und Jogaitė 2019)  Lithuania | Learning to whom children disclose experiences of harm caused by their parents’ or carers’ substance abuse. | In-depth semi-structured interviews | Children living with alcohol abusing caregivers in Lithuania | 23 | 8-18 | Alcohol misuse, three children of them who misused both alcohol and drugs | Received stigma;  Anticipated stigma;  Affiliate stigma;  Structural discrimination |
| 26. | (Trondsen und Tjora 2014)  Norway | Exploring how a web-based discussion forum designed for this particular group might provide support. | In-depth interviews (inductive, issue-focused approach ^Weiss, 1994^) | Adolescents with a mentally ill parent; using an online self-help group | 13 | 15-18 | Severe mental illnesses such as bipolar disorder, experienced psychotic periods, and attempted suicide | Affiliate stigma;  Structural discrimination |
| 27. | (van der Sanden et al. 2014)  Netherlands | Investigating the experiences of SBA and the burden; exploring coping with them. | Semi-structured interviews (inductive thematic content analysis) | Immediate family members (spouse, child, parent, sibling) of PWMI | 23  (20.7%  of them adult COPMI) | Participant ages 25-64; subgroup ages not reported | depression, bipolar, other mood disorder, personality disorder, ADHS/ADD, dissociative disorder, autism, schizophrenia/ psychotic disorder, addiction | Anticipated stigma |
| 28. | (van der Sanden et al. 2015)  Netherlands | Exploring the experiences of SBA. | Semi-structured interviews (thematic content analysis) | Immediate family members (i.e. spouses, children, siblings, and parents) of PWMI | 23  (20.7%  of them adult COPMI) | Participant ages 25-64 (M=44.3, SD=12.6); subgroup ages not reported | Depressive, bipolar, or other mood disorders (52.2%), personality disorder (17.4%), ADHD/ADD (17.4%), dissociative disorder (13%), schizophrenia or psychotic disorder (8.7%), addiction (4.3%) | Received stigma;  Anticipated stigma;  Affiliate stigma |
| 29. | (Wahl et al. 2017)  Germany | Examining the role of stigma in help seeking processes & encounters with formal & informal care; examining the relevance of stigma for COPMI | Semi-structured interviews (qualitative content analysis) | Parents with mental illness and their children | 31 (16 COPMI  and 15 parents) | 7-16 (M=10.94, SD=2.49) | Depressive episode, recurrent depressive disorder, comorbidity (personality disorder, addiction, impulsive-control disorder, anxiety/ panic disorder) | Anticipated stigma;  Affiliate stigma |
| 30. | (Widemalm und Hjärthag 2015)  Sweden | Identifying how COPMI perceive their situation, based on what they communicate on open Internet forums. | searching Swedish Internet forums (thematic analysis) | Forum posts written by individuals who reported that they had mentally ill parents | 197 different  Pseudonyms=indicator for 197 people | 13-49 (29 had stated their age) | Parental mental illness not further specified | Anticipated stigma;  Affiliate stigma;  Structural discrimination |
| *Quantitative studies* | |  |  |  |  |  |  |  |
| 31. | (Haverfield und Theiss 2016)  Canada | Examining severity of a parent’s alcoholism & family topic avoidance as factors associated with stigma perceptions; Assessing associations between perceived stigma & depressive symptoms, self-esteem and resilience | Online survey (multiple linear regression) | Adult children of alcoholics | 622 | 18-87 | Alcoholism | Received stigma;  Affiliate stigma |
| *Mixed methods* | |  |  |  |  |  |  |  |
| 32. | (Cogan et al. 2005a)  UK | Comparing coping with the family situation. | semi-structured interview, Adolescent Coping Scale (quant.: chi-square analysis, t-tests; qual.: interactive model) | Children of affectively ill parents and comparison group: children of “well” parents | 40 (20 COPMI, 20 non-COPMI) | 12-17 | Affective illness | Affiliate stigma;  Structural discrimination |
